# How do people with chronic pain choose their music for pain management? Examining the external validity of the cognitive vitality model

**DOI:** 10.3389/fpsyg.2022.969377

**Published:** 2023-02-09

**Authors:** Claire Howlin, Rosemary Walsh, Paul D'Alton, Brendan Rooney

**Affiliations:** ^1^Department of Biological and Experimental Psychology, School of Biological and Experimental Sciences, Queen Mary University of London, London, United Kingdom; ^2^Department of Arts and Sciences, University College London, London, United Kingdom; ^3^Psychology Department, St. Vincent's University Hospital, Dublin, Ireland; ^4^School of Psychology, University College Dublin, Dublin, Ireland

**Keywords:** pain, music, music listening, psychology, chronic pain, psychology, cognitive mechanisms

## Abstract

Music interventions for pain are more successful when patients choose the music themselves. But little is known about the attentional strategies used by chronic pain patients when choosing or using music for pain management, and the degree to which these attentional strategies align with the cognitive mechanisms outlines in the cognitive vitality model (CVM, a recently developed theoretical framework that outlines five cognitive mechanisms that mediate the analgesic effects of music for pain management). To investigate this question, we used a sequential explanatory mixed method approach, which included a survey, online music listening experiment, and qualitative data collection, with chronic pain patients (n=70). First, we asked chronic pain patients to name a piece of music that they would use to manage their chronic pain, and answer 19 questions about why they chose that particular piece of music using a questionnaire based on the CVM. Next, we asked chronic pain patients to listen to high energy and low energy pieces of music, to understand aesthetic music preferences and emotional responses at the group level. Finally, participants were asked to qualitatively tell us how they used music to manage their pain. Factor Analysis was completed on the survey data, and identified a five-factor structure in participant responses that was consistent with five mechanisms identified in the CVM. Regression analysis indicated that chronic pain patients choose music for pain management if they think it will facilitate *Musical Integration and Cognitive Agency*. *Musical Integration* refers to the degree to which the music can provide an immersive and absorbing experience. *Cognitive Agency* refers to having an increased feeling of control. At the group level, participants reported a preference for low energy music, and reported that they found high energy music more irritating. However, is it important to note that individual people had different music preferences. Thematic synthesis of patient responses highlighted how these processes mediate the analgesic benefits of music listening from the perspective of chronic pain patients, and highlighted the wide range of music used by participants for chronic pain management including electronic dance music, heavy metal and Beethoven. These findings demonstrate that chronic pain patients use specific attentional strategies when using music for pain management, and these strategies align with the cognitive vitality model.

## Introduction

The World Health Organization recommends arts-based interventions including music interventions as part of routine clinical care (Fancourt et al., 2019). This is particularly welcome for conditions that are not adequately managed by pharmacological treatments, such as chronic pain (Mainka et al., 2016). The international association for the study of pain defines pain as “An unpleasant sensory and emotional experience associated with, or resembling that associated with, actual or potential tissue damage” (Raja et al., 2020). This definition highlights that pain is a multi-dimensional experience, with cognitive, affective, and sensory components (Melzack, 1999), which means that pain management also needs to incorporate multi-dimensional and multi-disciplinary approaches alongside traditional pharmacological treatment, such as psychological therapies, tailored physiotherapy, and occupational therapy. The multi-disciplinary team can help patients to build physical strength, self-confidence and develop cognitive strategies to cope with extreme pain ‘flare-ups’. Music-based interventions provide new avenues to a wider range of supports for chronic pain patients; however, there is still much debate in terms of the way to optimize the introduction of music. For example, music interventions can be self-directed music listening (Gold and Clare, 2013) structured music therapy (Fitzpatrick et al., 2019), or as a cue to movement (Murrock and Higgins, 2009) which can indirectly improve pain management outcomes.

This use of music in interventions in routine pain management settings is supported by the results of several meta-analyses which indicate that music interventions reduce self-rated chronic pain (Garza-Villarreal et al., 2017), and can subsequently reduce the need for analgesic medication (Lee, 2016). The popularity of music interventions is also propelled by patients themselves who reportedly enjoy music listening and often use it as a way to relax (Fitzpatrick et al., 2019). One of the most appealing aspects of music interventions is that they are completely flexible and can quickly be adapted to meet the immediate needs of the patient. Additionally, music listening can be done at a time and place that is convenient for the patient (Robb et al., 2018; Fitzpatrick et al., 2019) and does not require additional hospital appointments or specialized equipment.

### What are the cognitive mechanisms that mediate the analgesic benefits of music listening?

**Figure 1:**
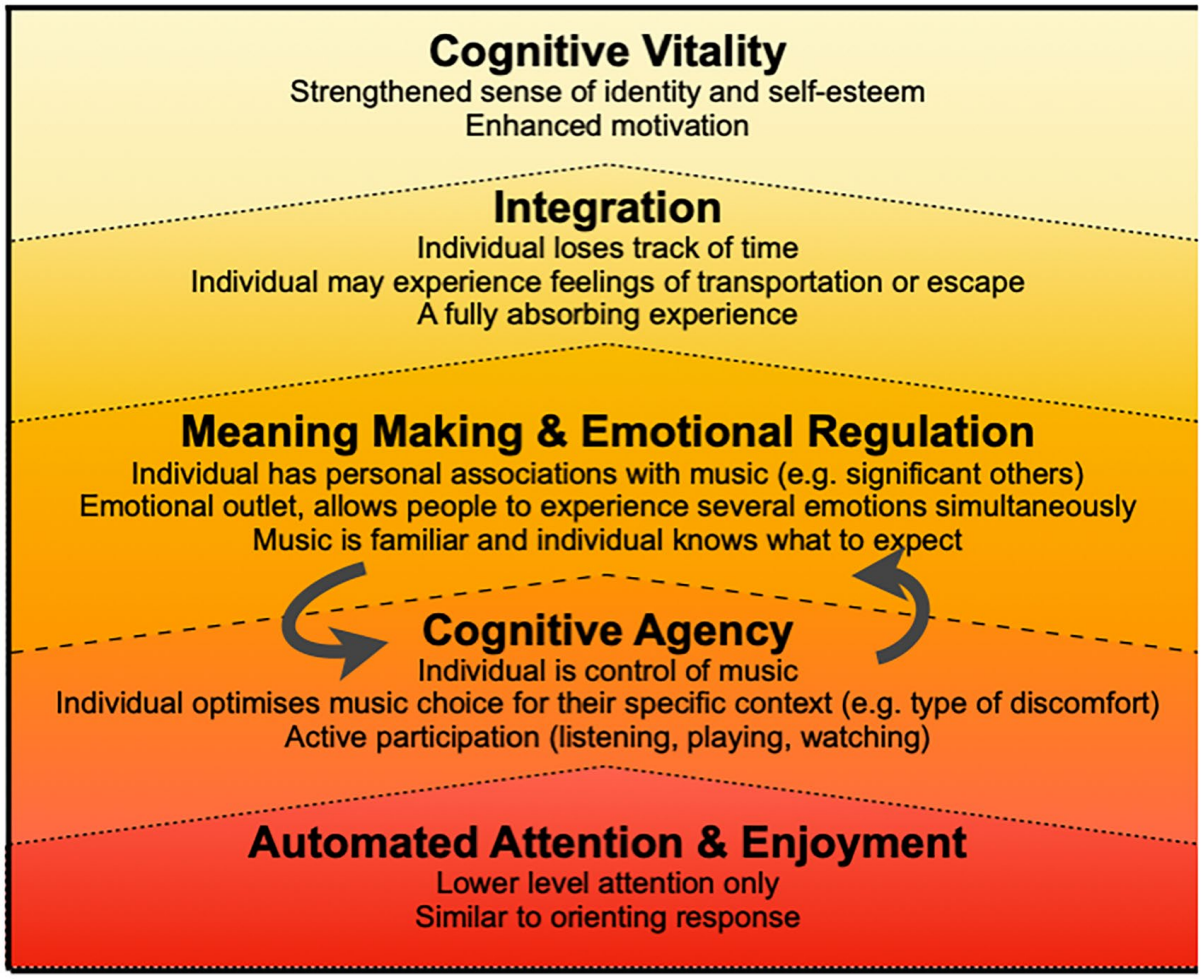
Cognitive mechanisms in cognitive vitality model. This depiction of the Cognitive Vitality Model includes details provided by chronic pain patients in the current study. The original version of the cognitive vitality model can be found in Howlin and Rooney (2020). Adapted with permission.

Self-chosen music is the greatest predictor of effective music-listening interventions for pain (Lee, 2016), and people with pain tend to choose music for pain management with different characteristics to what researchers and practitioners might think is optimal (Howlin and Rooney, 2021a). For example, although many experimenters and practitioners will select low-energy, instrumental music with gentle rhythms on behalf of a person with pain, the person with pain is more likely to choose more energetic, rhythmic music with lyrics. But little is known about the cognitive processes associated with such choice. In order to further refine music interventions and increase their overall therapeutic quality, there is a growing need to understand the cognitive mechanisms that mediate the beneficial effects of music-listening interventions (Keenan and Keithley, 2015; Lee, 2016). To this end, the Cognitive Vitality Model (CVM; Howlin and Rooney, 2020) provides a theoretical framework to understand the cognitive mechanisms involved in music interventions for pain management. The original Cognitive Vitality model is depicted in a previous publication (Howlin and Rooney, 2020), and a revised version based on the findings of the current study is presented in Figure 1. The CVM outlines five cognitive mechanisms that account for the different stages of cognitive engagement that involve that lead to the wellbeing effects observed in response to music (1) *Automated Attention* orientates the individual’s attention to the music and provides a lower-level distraction from pain. (2) *Cognitive Agency* is the way in which the person actively feels in control of the music, and uses self-directed music-listening strategies to actively engage with the music (3) *Meaning-Making and Enjoyment* is required to elicit personal reflection or aesthetic appreciation to deepen the level of engagement with the music, which motivates the person to keep listening and reduces the perceived effort involved in active listening. Meaning-making is key to emotional regulation processes as people can use the perceived meaning of music to reappraise their own thoughts and feelings, or because the person may have strong personal associations or memories with the music (e.g., going to concerts with friends, dancing at a wedding) that lead to a range of emotional responses. Eventually, after continued, uninterrupted engagement with the music (4) *Musical Integration* can occur, which means that the music is absorbed fully into the individual’s conscious experience on a cognitive, and emotional level. When musical integration occurs, the individual tends to lose track of time or feel like they have escaped, zoned out, or been transported to another place. Full absorption into the musical experience prevents the formation of competing constructions of reality that include the pain experience. Finally the person feels an enhanced sense of (5) *Cognitive Vitality* and cognitive energy which facilitates adaptive coping, an enhanced locus of control and a strengthened sense of self. Together these five mechanisms describe different states of cognitive engagement with music where people transition from lower level attention through to full absorption, and posits that higher levels of absorption in musical experiences elicit stronger wellbeing benefits. As indicated in Figure 1 automated attention forms the basis of deeper levels of engagement, and agentic, meaningful music experiences will be more likely to elicit the most benefits.

Empirical support for the CVM has been partly established in experimental studies (Howlin and Rooney, 2021b). Participants were presented with excerpts from music tracks to listen to while completing the cold pressor task, which involved submerging their hands into ice water until they felt a sense of discomfort. A unique experiment was devised to give participants perceived control of the music, when in fact it was pre-determined by the experimental design. When participants had the illusion that they were choosing the music, they demonstrated a higher pain tolerance compared to when they had no choice in the music. Additionally, self-rated enjoyment was a strong predictor of increased pain tolerance. Together these results provide evidence for the role of Cognitive Agency and Enjoyment in mediating the analgesic effects of music listening in the context of synthetic or experimental pain.

Although the CVM provides a framework for understanding the cognitive mechanisms that mediate the wellness benefits of music engagement, it is important to examine the external validity of the model. In the current study, we specifically focus on the perspective of chronic pain patients. This is particularly important because the psychological experience of chronic pain is different from the psychological experience of acute pain, because there is no sense of certainty that it will dissipate completely (Mitchell et al., 2007; Gold and Clare, 2013; Finlay, 2014). In order to understand the relevance of the CVM to chronic pain management, it is necessary to evaluate the model specifically with chronic pain patients.

### How does the CVM relate to the analgesic potential of self-chosen music?

One of the first things to explore is the degree to which the mechanisms outlined in the CVM relate to the *analgesic potential* of music selected by the patient. The analgesic potential of the music is the degree to which patients estimate that their music selection will be helpful with their pain management. Understanding the factors that contribute to the analgesic potential of music is important because self-selected music is the best predictor of a successful music intervention (Bradt et al., 2016; Garza-Villarreal et al., 2017, p.; Lee, 2016). The specific motivations patients have for choosing music is considered to be an important component in mediating the analgesic potential of music listening (Linnemann et al., 2015), because they increase patient motivation to maintain active cognitive engagement (Mitchell and MacDonald, 2006; Pothoulaki et al., 2008; Roy et al., 2008; Siedliecki, 2009; Good et al., 2010; Nguyen et al., 2010; Vaajoki et al., 2012; Finlay, 2014; Hsieh et al., 2014; Nagata et al., 2014; Linnemann et al., 2015). Previous research has identified that different pieces of music can be used to achieve the same analgesic benefits, a circumstance known as *functional equivalence* (Swaminathan and Schellenberg, 2015). What is not known is whether patients use the same cognitive strategies with different pieces of music to achieve these benefits. It is now time to explore how specific cognitive strategies relate to the analgesic potential of music. This will help to understand which mechanisms are more important in designing music-listening interventions for pain management.

Chronic pain patients’ preferences for specific intramusical features (e.g., music energy, tempo, key, or rhythmicity) is also an important factor to consider in music interventions. In line with current theories of emotional engagement with music (Juslin et al., 2008, 2014; Koelsch, 2010) the authors do not propose any specific music feature will be superior for pain management in a universal way, but instead, the analgesic benefits of music will be driven by patient preferences. This means that different types of music with different features can achieve the same analgesic benefits (Bradt et al., 2016; Lee, 2016; Garza-Villarreal et al., 2017), and is known as functional equivalence (Thaut, 2016), because different pieces of music can serve the same ‘function’. However, the key issue now is to identify and understand what the function of music listening is, in pain management contexts. Although many studies highlight the importance of using music to help people with pain to relax, a recent systematic compilation of music preferences for pain management, identified that people tend to choose music with a higher level of energy representing a range of valences (e.g., heavy metal music, electronic dance music, and upbeat pop music) compared music chosen by experimenters (e.g., typically classical, acoustic, and non-lyrical; Howlin and Rooney, 2021a). This undermines the idea that specific pieces of music will be more effective for pain management, and suggests that greater focus needs to be placed on the specific attentional and cognitive strategies used in music interventions. This study will help to disentangle the relative contributions between the cognitive strategies used in music interventions for pain management, and characterize chronic pain patients’ preferences for high-energy or low-energy music.

### Present study

The present study examined the nature of patient choice in music-listening interventions using an online survey and experimental design. The main aim of this study was to examine the external validity of the CVM. A questionnaire based on the mechanisms identified in the CVM was used to identify the degree to which patients’ motivations for choosing music for pain management aligned with the CVM. Additionally, a qualitative thematic analysis was used to gain a deeper understanding of patients’ experience of music listening for pain management. A secondary aim of this study was to assess chronic pain patients’ preference for different musical features, which was assessed using by asking chronic pain patients to provide aesthetic and emotional ratings for different music samples.

These aims were addressed with the following research questions:

RQ1a: Can the analgesic potential of patients’ self-chosen music be predicted by components of the CVM?

RQ1b: Do patient descriptions of music listening for pain correspond with the CVM?

RQ2: Do patients with chronic pain report any preferences in terms of the type of music that they find most beneficial for pain management?

## Materials and methods

### Study design

This study used an online survey and experimental design accessible by smart phone, tablet or home personal computer. A sequential explanatory mixed method approach was used to address the main research question (Ivankova et al., 2006) which involves two phases. The first phase involved a quantitative exploratory factor analysis of questionnaire responses and a subsequent regression analysis. The second phase involved a qualitative thematic analysis of patients’ responses to an open question. Mixed-methods sequential explanatory designs are particularly useful to capture the multi-dimensional aspects of pain experience and pain management (Melzack, 1999; Carr, 2009). The study design was approved by St. Vincent’s Hospital Research Ethics Board, and all chronic pain patients provided anonymous electronic consent in line with hospital ethics policy and General Data Protection Regulations.

### Patient recruitment

Patients with chronic pain were invited to participate in the study through pain management clinics in St. Vincent’s University Hospital, Dublin, and online through social media, using twitter and Facebook. The primary researcher attended weekly clinics and provided information leaflets for the study to 400 patients over 6 weeks. Patients named their diagnosis, which was then classified by the primary researcher according to the International Classification of Diseases 11 (ICD-11) definitions for chronic pain (Treede et al., 2015).

### Measures

#### Subjective pain

Participants rated their pain intensity and pain unpleasantness on mixed Numeric Rating Scales (NRS) using a pointer, before listening to the music. The 100-point intensity scale had three anchor points ‘no pain’ (0), ‘moderate pain’ (50), and ‘worst pain imaginable’ (100). The 100-point unpleasantness scale ranged from ‘not unpleasant’ (0) to ‘extremely unpleasant’ (100). Numeric rating scales (NRS) are considered the gold standard for measuring patient’s subjective feeling of pain intensity and pain unpleasantness, because they are more sensitive than other self-report measures that treat pain as a unidimensional construct (Breivik et al., 2008).

#### Analgesic potential of self-chosen music

Patients were asked to estimate how much their chosen music piece would help to reduce their pain on a continuous Likert scale ranging from 0 ‘it would not help at all’ to 100 ‘It would help a lot’.

#### Wellbeing

The CASP-19 Quality of Life Scale was used to measure wellbeing based on four domains; Control, Autonomy, Self-realization, and Pleasure (CASP; Hyde, et al., 2003). The CASP-19 includes 19 items which are scored on a 4-point Likert scale ranging from 0 ‘never’ to 3 ‘often’. Scores range from 0 to 57 with higher scores indicating a higher quality of life.

#### Cognitive vitality questionnaire

Twenty-one items were created for the Cognitive Vitality Questionnaire based on the CVM (Howlin and Rooney, 2020). The initial items were constructed based on 75 journal articles, which included patient qualitative reports, neuroscientific research, clinical trials, and psychology experiments. Each item provided a statement that described a reason for choosing a piece of music, and participants were asked to rate the degree to which they agreed or disagreed with each statement. Participants responded on a Likert scale ranging from 0 ‘strongly disagree’ to 100 ‘completely agree’. Nineteen items were included in the initial questionnaire and factor analysis, and the 16 items that contributed to the final factor structure were kept for the final analysis. The items included in final questionnaire can be seen in Table 1.

**Table 1:** Initial 19 items included in cognitive vitality questionnaire.

Factor 5 Musical Integration	This song produces a whole-body experience
	I lost track of time as I am listening to music
	Listening to this song gives me an opportunity to be myself
	This song gives me mental strength
Factor 4 Personal Meaning	The lyrics in this song are meaningful to me
	This is a beautiful piece of music to me
	Most people would agree with my opinion of this song
	This song does not remind me of any specific memories*
	Listening to this song reminds me of good times*
Factor 3 Motivation	Overall how much does this song make you want to move
	Overall how much are you energized by this song
Factor 2 Cognitive Agency	I have a specific reason that I would listen to this song
	I do not think this was a good choice of song
Factor 1 Attention and Enjoyment	Overall how much were you bored by this song? A
	Overall how much did you enjoy this song?
	This is mainly just Background music
	This song does not capture my attention
	Overall, how much were you distracted by this song?
	This song would take over my thoughts effortlessly*

Nineteen items were included in the original cognitive vitality questionnaire. *items did not load onto the factor structure of the questionnaire so data from these questions were not included in the final analysis, and should not be used.

#### Musical emotional response

Participants completed the short version of the Geneva Emotional Musical Scale (GEMS-9; Zentner et al., 2008) to evaluate emotional response to each piece of music. The GEMS presents a nine-dimensional emotional structure to account for emotional responses to music. Each factor is independent of the other factors which has been established with exploratory and confirmatory factor analyses and the model provides a better account of emotional responses to music than non-domain-specific emotional models (Zentner et al., 2008).

### Music stimuli

A pilot study was used to select 6 pieces of music which were used in a previous lab-based experiment (Howlin and Rooney, 2021b), and the same pieces of music were used in the current study. For the pilot study six people provided familiarity ratings on a continuous rating scale from 0 ‘not familiar at all’ to 10 ‘extremely familiar’ (See Table 2). Familiarity was controlled for to reduce the likelihood that people would provide aesthetic ratings and emotional responses based on their personal familiarity with the music rather than the audio features, because familiarity presents enhanced opportunities for emotional engagement and enjoyment for the listener (Good et al., 2010; Brattico and Pearce, 2013), independently of the music features. The Spotify Audio features of danceability, energy, and tempo were used to control for different audio features, based on the results of a previous study that identified that people tend to choose music with significantly higher levels of danceability, energy and lower levels of instrumentalness compared to music chosen by experimenters (Howlin and Rooney, 2021a). These Spotify audio features were used based on the results of a previous study that demonstrated that people tended to choose music that was significantly higher in energy. Music with high levels of energy, danceability, and tempo was labeled as *High Energy*, and music with low levels of energy, danceability, energy, and tempo was labeled as *Low Energy*. All songs that were commercially available without lyrics had a mean familiarity rating of 3 or lower. This resulted in three Low-Energy music pieces: *Sleeping Music* by Deep Sleep Music Collective, *This Isn’t You* by Kyle Dixon, and *Danger of Hell* by Thomas Newman; and three High-Energy music pieces: *Solero* by Sons of Maria, *Lighthearted* by Deep Chills, and *The Balance* by Moses Boyd.

**Table 2:** Music Stimuli and Familiarity Ratings from Pilot Study.

**Title**	**Artist**	**Energy**	**Danceability**	**Familiarity Rating**
				*M* (S*D*)
Low Energy Music				
*Sleeping Music*	Deep Sleep Music Collective	0.00	0.13	3.0 (3.2)
*This Isn’t You*	Kyle Dixon	0.02	0.13	2.6 (3.2)
*Danger of Hell*	Thomas Newman	0.01	0.19	2.2 (2.7)
High-Energy Music				
*Solero*	Sons of Maria	0.94	0.80	2.6 (2.6)
*Lighthearted*	Deep Chills	0.43	0.77	1.8 (1.3)
*The Balance*	Moses Boyd	0.84	0.61	1.0 (1.2)

These six pieces of music were selected from a wider pool of 20 songs which were selected based on the Spotify audio features of Energy, and Danceability. All of these pieces of music were instrumental without lyrics. M = Mean, SD = Standard Deviation. Energy and Danceability are absolute values taken from the Spotify Developer website that run from a minimum of 0 to a maximum of 1. Familiarity was rated by 6 human participants in a separate pilot study and is scored from a minimum of 0 to a maximum of 10.

### Procedure

Participants completed the online survey at a time and location that was convenient for them. Each participant listened to six pieces of music which were presented in counterbalanced order. For each piece, participants completed the GEMS-9; (Zentner et al., 2008) and rated the music in terms of enjoyment, boredom, and irritation. Once the music-listening trials were complete participants were then asked to name any song that they thought would be good to help manage their pain, and rate the analgesic potential of their chosen song. Next, participants completed the Cognitive Vitality Questionnaire in response to the song that they had chosen. Finally, in an open question, participants were asked if they had “anything else to add about listening to music when you have chronic pain.” The experiment took approximately 45 min to complete.

### Data analysis

Exploratory factor analysis was conducted on participants responses to the cognitive vitality questionnaire to examine the factor structure of the responses. Regression analysis was then used to examine if scores for each factor could predict ratings for how effective the participants thought the self-chosen song would be for pain management. This allowed us to examine which factors were most important in mediating the analgesic benefits of music listening from chronic pain patients perspective. The qualitative analysis was used to explore the quantitative results in more detail.

## Results

### Patient characteristics

Seventy patients with chronic pain completed the study. Nine participants were recruited from pain management clinics in St. Vincent’s University Hospital, Dublin. Additionally, 61 participants were recruited to participate *via* social media. The entire sample of 70 patients had an age range of 18–70 (*M* = 43.12, SD = 12.09), and was comprised of 56 females, 13 males, and 1 transgender person. The sample consisted of 26 (37.1%) patients with primary chronic pain, 17 (24.3%) patients with chronic musculoskeletal pain, 7 (10.0%) patients with neuropathic pain, 6 (8.6%) patients with multiple independent diagnoses, 6 (8.6%) patients with chronic visceral pain, 4 (5.7%) patients with chronic postsurgical and posttraumatic pain, 3 (4.3%) patients with chronic headache and orofacial pain, and 1 (1.4%) patient with chronic cancer pain. Pain intensity scores ranged from 0 to 10 with a mean of 5.43 (SD = 1.98). Pain unpleasantness scores ranged from 0 to 9 with a mean of 5.20 (SD = 1.99). Wellbeing scores measured using the CASP-19 ranged from 7 to 54 and patients reported a mean wellbeing score of 28.31 (SD = 9.48).

### (RQ1a) To what extent can the analgesic potential of patients’ self-chosen music be predicted by components of the CVM?

The main research question was examined using a sequential explanatory mixed methods approach. This approach allows us to conduct a quantitative analysis followed by a qualitative analysis in order to gain a greater understanding of the quantitative findings (Ivankova et al., 2006).

The goal of the quantitative phase was to identify the degree to which the mechanisms outlined in the CVM relate to the *analgesic potential* of music selected by the patient. In order to achieve this the quantitative analysis was conducted in two parts. First, an exploratory factor analysis was conducted to examine the factor structure of the questionnaire and quantify how patient responses corresponded with the cognitive mechanisms identified in the CVM. Second, a regression analysis was conducted to examine how patient scores for each factor of the cognitive vitality questionnaire predicted the analgesic potential of the patients chosen song.

#### Factor structure of cognitive vitality questionnaire

An exploratory factor analysis was conducted to examine the factor structure of the cognitive vitality questionnaire, to examine the factor structure of the questionnaire, and to identify the patterns that emerge in patient’s agreement with the cognitive mechanisms identified in the CVM. Initially, a principal components analysis was completed on all 21 items in the Cognitive Vitality Questionnaire (CVQ). The Kaiser-Meyer-Olkin measure of sampling adequacy was 0.75, which indicated that we achieved sampling adequacy (Kaiser, 1974). Bartlett’s test of sphericity (Bartlett, 1954) indicated that there were sufficient intercorrelations between the items to justify the application of Exploratory Factor Analysis [*χ*^2^(171) = 561.69 *p* < 0.001]. On examination of the scree plot, and the eigenvalues, a five-factor solution was determined as the most appropriate to fit the model. This decision eliminated one factor that had an eigenvalue greater than one; however, this factor only included two negatively worded items, that were not otherwise related, and it appeared to the research team that it was more likely that it was the wording of the item that was causing people to rate them similarly rather than an underlying construct. Instead, the remaining five factors were considered to represent the latent constructs outlined in the CVM, and a common factor analysis was completed. Principal axis factoring, with a Promax rotation with Kaiser Normalization was used to account for the fact that the data was negatively skewed (as patients were positive overall) and the small sample size (Fabrigar et al., 1999). This method was determined as appropriate because it does not require a large sample size and makes no assumptions about the underlying distributions of the data (Watkins, 2018). Items with a loading of less than 0.4 were removed. Each factor was named based on the content of the final items included in each factor, in line with the proposed factors of the CVM.

The five-factor solution was examined for adequacy. Each factor was loaded by a minimum of two items (see Table 3 for eigenvalues and communalities for each factor), and each item was cleanly loaded onto only one factor. Following Factor rotation factor 1 accounted for 10.57% of the common variance, factor 2 accounted for 6.86% of the common variance, factor 3 accounted for 9.06% of the common variance, factor 4 accounted for 7.65% of the common variance, and factor 5 accounted for 29.91% of the common variance. In total the five factors accounted for 64.12% of the variance in agreement scores. The factor correlation matrix indicated that the factors were correlated at less than 0.3 except for factor 1 and factor 2 which were correlated at 0.54, and factor 1 and factor 4 which were correlated at 0.39. Given these results, the five-factor solution was accepted as an adequate structural representation of the Cognitive Vitality Questionnaire (CVQ). However, it was noted that factor 2 and factor 3 would benefit from additional items.

**Table 3:** Factor analysis table for cognitive vitality questionnaire.

	F5	F4	F3	F2	F1	Communality
CVQ18 This song produces a whole-body experience	0.879					0.657
CVQ17 I lost track of time as I am listening to music	0.780					0.747
CVQ20 Listening to this song gives me an opportunity to be myself	0.640					0.462
CVQ13 This song gives me mental strength	0.619					0.582
CVQ2 Overall how much were you bored by this song?					0.861	0.790
CVQ1 Overall how much did you enjoy this song?					0.690	0.787
CVQ10 This is mainly just Background music					0.642	0.557
CVQ16 This song does not capture my attention					612	0.501
CVQ3 Overall, how much were you distracted by this song?					0.561	0.417
CVQ5 Overall how much does this song make you want to move			0.949			0.635
CVQ4 Overall how much are you energized by this song			0.721			0.613
CVQ9 The lyrics in this song are meaningful to me		0.825				0.440
CVQ15 This is a beautiful piece of music to me		0.565				0.615
CVQ14 most people would agree with my opinion of this song		0.435				0.440
CVQ11 I have a specific reason that I would listen to this song				0.631		0.429
CVQ12 I do not think this was a good choice of song				0.574		0.517
Eigenvalue	5.70	1.45	1.72	1.30	2.01	
%of Total Variance	29.30	7.65	9.06	6.87	10.57	
Total Variance					64.12%	

F1 Attention and Enjoyment, F2 Cognitive Agency, F3 Motivation, F4 Personal Meaning, F5 Musical Integration and Vitality.

Factor 1 was labeled *Attention and Enjoyment* and refers to the way in which any music will automatically grab people’s attention and that enjoyment or reward responses are implicit in the automatic engagement.

Factor 2 was labeled *Cognitive Agency* and refers to the specific reasons people have when choosing a piece of music to listen to which can increase the patient’s locus of control.

Factor 3 was labeled *Motivation* and is a subcomponent of the mechanism called cognitive vitality and refers to the *motivation* that people can feel as a result of personal music listening.

Factor 4 was labeled *Personal Meaning* and refers to the personal connection people have with and may remind them of a significant person or event in their life or be an important part of their identity.

Finally, factor 5 was labeled *Musical Integration and Vitality* which refers to how music is integrated into the person’s conscious awareness on a cognitive and emotional level. Musical Integration relies on absorption in the music and is characterized by losing track of time.

These factors corresponded with the factors outlined in the CVM, with some minor adjustments; enjoyment overlapped more with automated attention processes rather than with meaning-making as proposed in the original CVM. This suggests that attention and enjoyment are more tightly interlinked from a chronic pain patient’s perspective, and meaning and enjoyment may be separate processes. Additionally, some aspects of vitality were grouped more closely to integration, whereas aspects of vitality related to motivation loaded onto an independent factor. The high level of agreement from participants across the items suggests that these factors are a strong representation of the patients’ intentions for analgesic music listening, and corresponds with the CVM. The implications of these variations in the boundaries between the factors are considered further in the discussion section.

#### Relationship between CVQ factors and analgesic potential of patient chosen music

**Figure 2:**
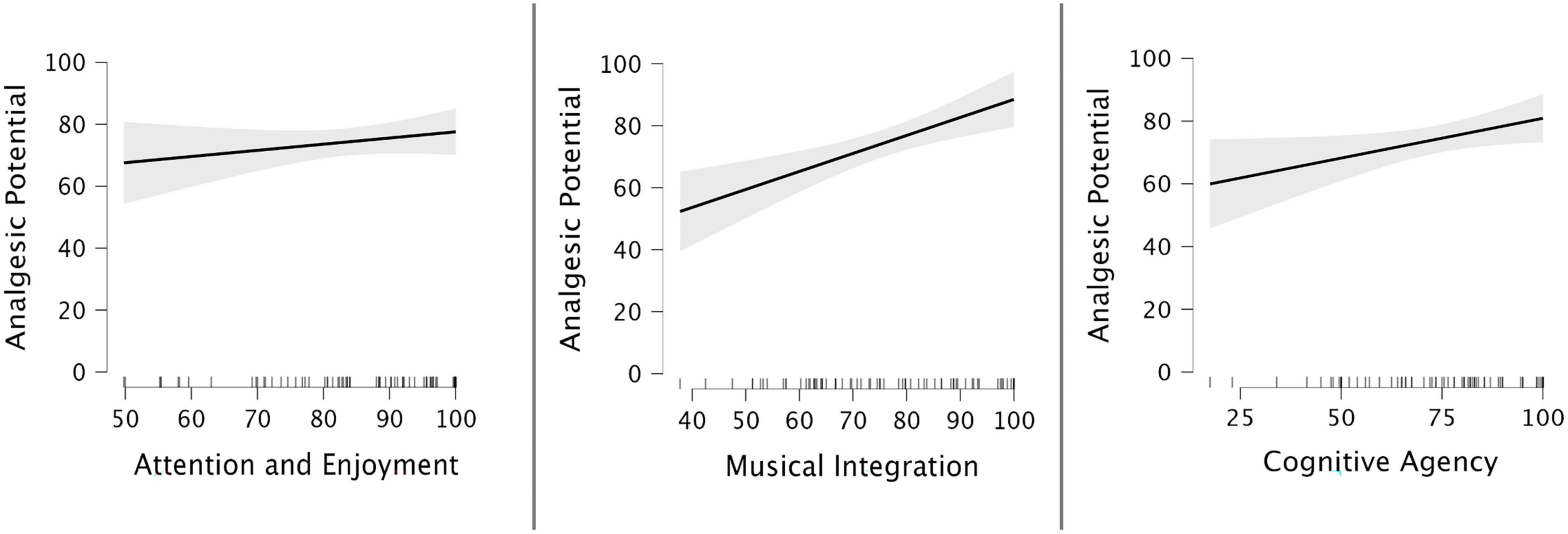
Regression plots of marginal effects. This diagram shows the individual marginal effects of each factor included in the linear regression model Attention and Enjoyment, Musical Integration, and Cognitive Agency. The *y*-axis indicates the analgesic potential scale from 0 to 100. The steeper slope for Musical Integration and Cognitive Agency illustrates that participants consider it to be more important when they are choosing their music than Attention and Enjoyment.

Next, we examined how patient scores for each factor of the cognitive vitality questionnaire were related to the analgesic potential of the patients’ chosen song. Once each factor was identified, mean scores were calculated for each factor. Each factor was then correlated with the *analgesic potential* of the music. To account for the marginal skewness in the data non-parametric Spearman’s correlations were used. Overall higher levels of agreement with each factor were positively related to how much the music would help to reduce their pain experience. Moderate positive correlations were found between the analgesic potential rating and the factors *Musical Integration r_s_*(69) = 0.682, *p* < 0.001, *Automated Attention* and *Enjoyment r_s_*(69) = 0.530, *p* < 0.001, and *Cognitive Agency r_s_*(69) = 0.492, *p* < 0.001. Weak positive correlations were found between the Benefit for Pain rating and Motivation *r_s_*(68) = 0.317, *p* < 0.01 and *Meaning-Making r_s_*(68) = 0.318, *p* < 0.01. The strength of the correlations was used to select which factors to include in a regression analysis. The three factors (*Musical Integration*, *Automated Attention* and *Enjoyment*, *and Cognitive Agency*) that were moderately correlated with the *analgesic potential* rating were then entered into a linear regression analysis to predict the outcome variable of analgesic potential. The regression model was significant, *F*(1, 64) = 39.85, *p* < 0.001, *R*^2^ = 0.559. The analgesic potential was significantly predicted by *Musical Integration*, *ß* = 0.67, *t*(63) = 6.69, *p* < 0.001; SE = 0.10, 95% CI [0.47, 87], and *Cognitive Agency*, *ß* = 0.24, *t*(63) = 2.96, *p* < 0.01; SE = 0.08, 95% CI [0.08, 41]. This indicates that *Musical Integration* was the best predictor as it had the highest beta co-efficient of 0.67, followed by *Cognitive Agency,* with a beta co-efficient of 0.24. This means that for every 1-unit increase in Musical Integration scores for a chosen song, the analgesic potential of that song increased by 0.67. For every 1-unit increase in Cognitive Agency scores for a chosen song, the analgesic potential increased by 0.24. This suggests that Chronic pain patients think that the degree to which a song will elicit Musical Integration is the most important factor leading to subsequent analgesic benefits, but they also think that their specific music choices are an important component in achieving music analgesia (Figure 2).

### (RQ1b) In what way do patient descriptions of music listening for pain correspond with the CVM?

The goal of the qualitative phase was to help understand why *Musical Integration* and *Cognitive Agency* were the mechanisms most closely linked to whether patients thought their chosen song would be beneficial for pain management. Qualitative analysis of patient responses to an open-ended question was completed using thematic synthesis and the results are displayed in Table 4. Forty-four patients responded to the open question. The thematic synthesis strategy was developed by the research team and involved three stages (Thomas and Harden, 2008). The first reviewer coded the text line by line according to its meaning or content. Next, codes were grouped together based on their similarity, so as to develop descriptive themes. Finally, descriptive themes were then grouped together to form analytical themes. When developing the analytical themes the researchers focused on descriptions related to *Musical Integration* and *Cognitive Agency* highlighted by patients as important in the quantitative phase. Additionally, two other analytical themes were developed based on patient responses. A second reviewer performed a credibility check on all of the descriptive themes and analytical themes and agreed with 90% of the coding decisions made by the first reviewer. After discussion between the reviewers, some codes were amended and both reviewers were in 100% agreement. Four themes were developed, Musical Integration, Cognitive Agency, Emotion Regulation, and Optimal Arousal.

**Table 4:** Results of qualitative analysis.

Analytical theme	Descriptive theme	Codes
Musical Integration	Escape From Reality	Escape beyond the music
		Getting lost
		Transportation
	Absorption	Particularly involving
		Zone out
	Forget about pain	Forget about troubles
		Take away thoughts of pain
Cognitive Agency	Individuality	Self-chosen music
		Unique music preference
		Specific genre or artist preferences
	Self-strengthening	Feeling more than the illness
		Lost without music
		Feel for a while you are just like everyone else
	Active Participation	Playing music
		Music Lessons
		Watching music videos
Emotion Regulation	Personal Meaning	Lyrics
		Sentimental Meaning
		Reminder of specific people
	Familiarity	Expecting the beat
		Familiar
	Emotional Regulation	Uplifting
		Emotional outlet
		Wallow too much
		Coping strategy
		Amplify emotion
		Experience different emotions simultaneously
Optimal Arousal	Relaxation	Calm music
		Peaceful atmosphere
		Meditation
		Complex music with layers
	Physical Motivation	Energetic music
		Music with a beat
		Music for Movement
	Match Music to outcome	Different music for different levels of pain
		Upbeat music for movement
		Dreamy instrumental music for relaxation
		Relaxing music can be boring

#### Musical integration

The theme of *Musical Integration* included the descriptive themes of *absorption*, *transportation*, *escape from reality*, and *forget about pain*. Participants described how music could be used as a mental escape from pain to transport them out of their current and subsequent experiences. As described by patient 30 “*I use music to transport myself out of this world*.” The benefits of being transported away were attributed to emptying the mind of pain-related thoughts even though the physical sensation may be present as described by patient 34 “*So, it’s more about Not Thinking. Pain may still be there but subdued*.” Musical Integration was described as having long-lasting effects after music-listening due to an enhanced mood. This was highlighted by several patients including patient 3 “*It’s an escape, not just into the music but beyond afterward with the effects on my mood directly lessening my pain*.” Participants provided several descriptions of musical engagement that were consistent with absorption, e.g., “*zoned out*” and linked these to feeling disconnected from their current environment including physical pain sensations and thoughts about pain.

#### Cognitive agency

The theme of *Cognitive Agency* was comprised several descriptive themes including *independence, self-strengthening*, and *active engagement.* Participants described the importance of engaging in an activity that was personally important to them and emphasized the importance of music in their life more generally. Some participants expressed an independence in their music-listening preferences habits and emphasized that they thought the way that they used music to manage pain was quite specific to them and would be unlikely to benefit other people. For example, participant 10 reports “*This works for me and probably would not work for others*.” Similarly, participants described that having an opportunity to express themselves in a way that was independent of their pain was an important factor in identifying the different parts of themselves that co-exist alongside their pain identity. This was highlighted by participant 6 “*It provides an anchor and reminds me that I am more than my illness.*” Additionally, several participants reported ways in which they actively engage with music, either by taking music lessons or by selecting specific soundtracks.

#### Emotion regulation

The theme of *Emotion Regulation* encompassed the descriptive themes of *Personal Meaning*, *Emotion Regulation*, and *Familiarity*. Participants reported that music can be used either to elevate mood or as an emotional release which may involve crying or laughing. Familiar music with meaningful lyrics was considered beneficial for emotion regulation by several participants. Two participants identified that they use music which reminds them of a significant loved one which brings them great comfort. Participant 17 summarized how music with a sentimental meaning made them feel happier: “*And sometimes it’s nice just to listen to songs with sentimental meaning to bring me back to a happier time or place in my mind.*” However, participants were divided on the degree to which emotional regulation can actually lessen the physical sensation of pain. While some participants reported that music can directly help their pain, other participants reported that music had no impact on their physical sensation of pain and was only useful for emotional regulation.

#### Optimal arousal

The theme of *Optimal Arousal* included the descriptive themes of *Attention and Enjoyment*, *Motivation*, *Relaxation*, and *Negative Effects*. The overwhelming commentary that came from participants reflected the importance of matching music energy to the desired outcome for the patient. Across the board patients highlighted the importance of matching the music to the participants’ pain level, and the type of task they wished to engage in. Participant 36 summarizes how different types of music can be used for different activities: “*Sometimes I need energetic music I can sing along to while I try do some housework. Then to relax something more complex with various layers to it that I can close my eyes and concentrate on and follow an instrument.*” An apparent paradox was identified by several participants that they liked music that could simultaneously energize them and help them to feel relaxed by relieving their tension. For example, participants 31 describes how they like *‘soothing music*’ that gets the ‘*circulation going*’ Participants had very unique perspectives in terms of which features they thought would be most effective, and no features were considered universally effective by the patient group. For example, some chronic pain patients preferred strong beats, while others preferred meditative or string music. It is important to note that several patients reported that they would find any music irritating during times of severe pain, as highlighted by participants 61 “*in an episode of severe pain I would feel irritated listening to even my favorite music so was unable to choose a song to use as a therapy*.”

### (RQ 2) Do patients with chronic pain report any preferences in terms of the type of music that they find most beneficial for pain management?

**Figure 3:**
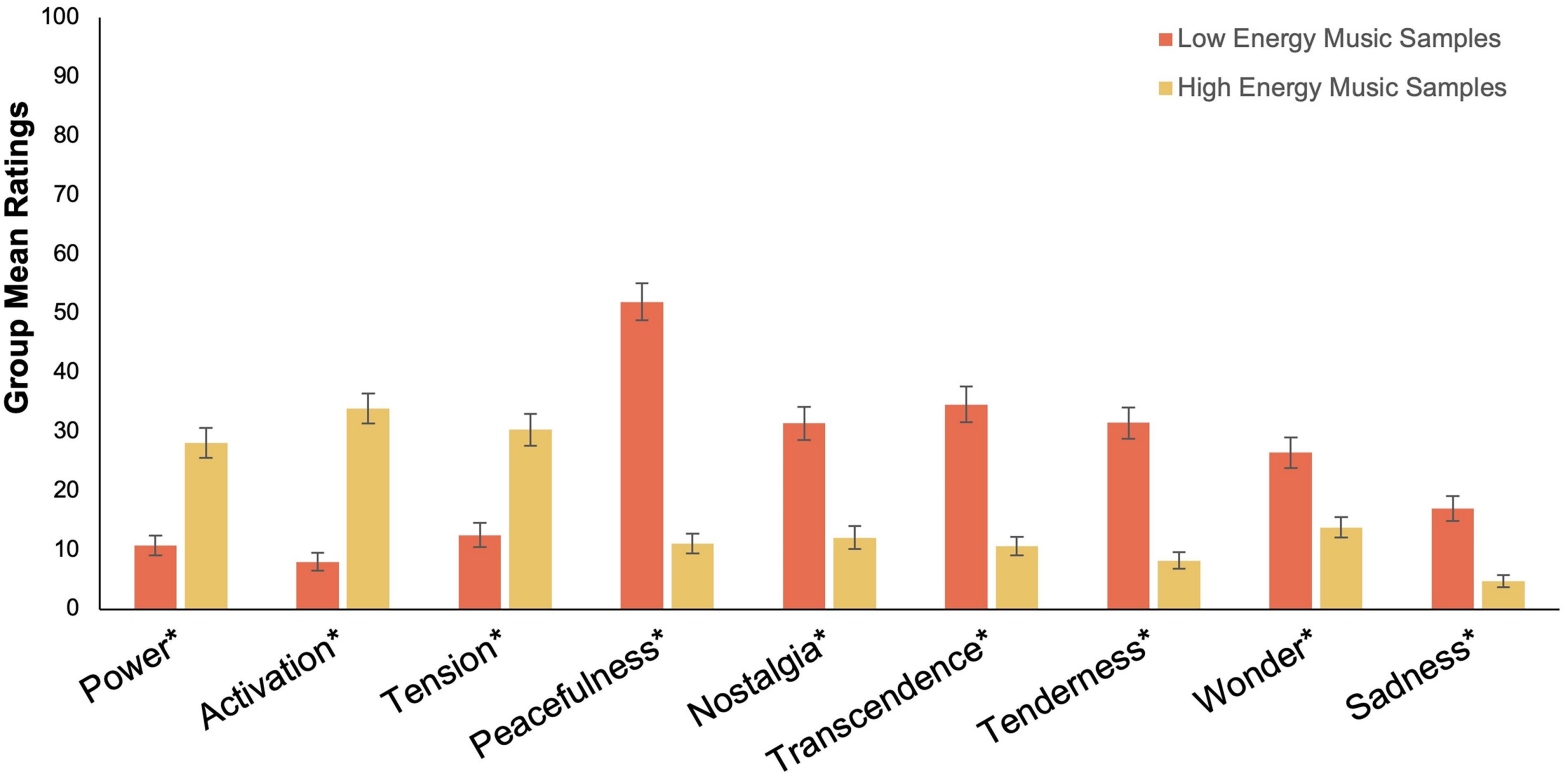
Group Mean Ratings for Emotional Responses For High Energy and Low Energy Music. This graph illustrates that chronic pain patients had significantly different emotional responses to high energy music compared to low energy music. Emotional ratings were provided on an amended version of the Short version of the Geneva Emotional Musical Scale (GEMS-9), using a scale of 1–100. The *y*-axis indicates the possible range of scores from 1 to 100. Error bars denote one standard error around the mean. Comparisons between the group mean scores of emotional responses were made using paired t-tests. *Significant at the 0.001 alpha level.

Finally, patient preferences in music for chronic pain management were explored. Paired t-tests were used to demonstrate that patients rated music that was classified as low energy (e.g., ‘relaxing music’) as significantly more enjoyable; *t*(69) = 3.57, *p* < 0.001 95% CI [6.32, 22.39] with a significantly higher analgesic potential; *t*(69) = 5.16, *p* < 0.001 95% CI [11.25, 25.42] and significantly less irritating *t*(69) = 4.86, *p* < 0.001 95% CI [9.22, 22.10] compared to music with high levels of energy (e.g., ‘motivating music’). However, while these results demonstrate that patients rated low-energy music as more enjoyable, more helpful for reducing pain, and less irritating compared to high-energy music, overall it is important to note that the wide confidence intervals here indicate a high degree of variation between individual patients. No difference was found in ratings of boredom between high-energy music and low-energy music. Additionally, we compared patients’ emotional responses between the two types of music. Low-energy music was rated as inducing significantly higher levels of *Wonder*, *Transcendence*, *Tenderness*, *Peacefulness*, *Sadness*, and *Nostalgia* responses compared to high-energy music (See Figure 3). High-energy music was rated as inducing significantly higher levels of *Power*, *Activation*, and *Tension* responses compared to low-energy music (See Figure 3). These results indicate that chronic pain patients had different patterns of emotional responses to high-energy music compared to low-energy music.

Although different levels of enjoyment were identified between the two different types of music, we also wanted to investigate the role of enjoyment overall and examine how it relates to the analgesic potential in patients’ chosen music. To account for the marginal skewness in the data a non-parametric Spearman’s rho correlation was calculated. A moderate positive correlation was found between patient ratings of enjoyment and patient ratings for the analgesic potential of their chosen song, *r_s_*(69) = 0.497, *p* < 0.001. Subsequently, a linear regression was calculated and identified that self-rated enjoyment was a significant predictor of how helpful people thought their chosen song was for pain management, *F*(1, 67) = 11.57, *p* < 0.001, *R^2^* = 0.147. This indicates that while enjoyment is a significant predictor of the likelihood of pain reduction, on its own it only accounts for 14.7% of the variance.

## Discussion

Until now, the degree to which the CVM corresponds with chronic pain patients’ experience of music listening as a pain management strategy, was untested. The main aim of this study was to explore the degree to which the analgesic potential of patients’ self-chosen music can be predicted by components of the CVM. Overall, the findings demonstrate that patients rated the factors of *Musical Integration* and *Cognitive Agency* as the most strongly linked to the analgesic potential of their chosen song. This means that different pieces of music are being used by people with chronic pain to facilitate cognitive strategies that correspond with cognitive agency and musical absorption. This result was based on a quantitative analysis that was conducted in two parts. First, an exploratory factor analysis was conducted to examine the factor structure of the questionnaire and quantify how patient responses corresponded with the cognitive mechanisms identified in the CVM. The factor analysis demonstrated that the pattern of patient responses corresponded with five factors that represented the five cognitive mechanisms outlined by the CVM. This suggests that chronic pain patients are largely in agreement with the cognitive mechanisms outlined in the CVM. While patients may differ in the specific music that they choose for pain management, it seems that there is relatively strong agreement in terms of why patients are choosing the music. Second, a regression analysis was conducted to examine how patient scores for each factor of the cognitive vitality questionnaire predicted the analgesic potential of the patient’s chosen song. The regression analysis demonstrated that the analgesic potential of the music was most strongly predicted by the factors of Musical Integration and Cognitive Agency. This result is in line with the CVM which emphasizes the importance of Musical Integration and Cognitive Agency in facilitating an enhanced sense of self and subsequent vitality as a result of music listening for pain management. Patients’ tendency to acknowledge the relationship between the cognitive mechanisms of Cognitive Agency and Musical Integration and the music’s analgesic potential suggests that patients have a conscious awareness of these two mechanisms. In this instance, patients recognized that their music choice was motivated by specific reasons, which gives participants a chance to exert their individual autonomy and subsequently enhance their internal locus of control. Additionally, patients’ interpretation of Musical Integration on the questionnaire was tightly related to a strengthened sense of self and mental energy. This suggests that the mechanism of musical integration is not readily separable from the mechanism of cognitive vitality from the chronic pain patient’s perspective and may be more tightly interwoven than initially outlined by the model. Similarly, chronic pain patients were more likely to consider enjoyment as a component of attention rather than meaning-making as proposed by the initial model.

These discrepancies between the initial proposed model, and chronic pain patients’ ratings, need to be considered in further detail, both methodologically and theoretically. Factor 4, Personal Meaning, is intended to reflect meaning--making processes related to emotion regulation (Meyer, 2008). Given that chronic pain patients specified the importance of emotion regulation in their own words, it may be beneficial to try and create items that have more accessible everyday language to reflect meaning-making processes. Factor 1 automated attention and enjoyment reflects a lower-order cognitive process, and would not be expected to elicit analgesic effects in isolation (Howlin and Rooney, 2020) so it is not surprising that chronic pain patients did not consider this as one of the most important factors when choosing their music. Surprisingly, a new factor of Motivation emerged in the factor analysis and needs to be explored further. The items in Motivation were initially intended to group with Cognitive Agency, but the fact that they grouped as an independent factor and that participants identified in their own words that they like to choose music with optimal arousal suggests that chronic pain patients are aware of specific strategies that they use to choose music. Further clarification of the exact boundaries between each factor, and the order in which they occur could be determined using a confirmatory factor analysis, with additional items included in more accessible language. Nonetheless, the high-level agreement from participants across the items suggests that these items and factors are a strong representation of the patients’ intention for analgesic music listening, which demonstrates that the CVM has reasonable external validity from chronic pain patients’ perspective.

### Patient descriptions of music listening for pain that correspond with the CVM

Patient descriptions of the benefits of music listening were used to explore the questionnaire responses in more detail using qualitative analysis. This helped to gain a greater understanding of the quantitative results, and to provide more insight into the specific reasons patients have for using music for pain management. The specific reasons for music listening are an important component of music-listening interventions (Linnemann et al., 2015), since they increased patients’ motivation to maintain active engagement and sustain the musical experience (Mitchell and MacDonald, 2006; Mitchell et al., 2008; Pothoulaki et al., 2008; Roy et al., 2008; Nilsson, 2009; Siedliecki, 2009; Good et al., 2010; Nguyen et al., 2010; Vaajoki et al., 2012; Finlay, 2014; Hsieh et al., 2014; Nagata et al., 2014; Linnemann et al., 2015). Four themes were developed using thematic synthesis, which were Musical Integration, Cognitive Agency, Emotion Regulation, and Optimal Arousal. Since the two mechanisms of Musical Integration and Cognitive Agency were related to the analgesic potential of patient’s self-chosen music, this will now be discussed in more detail.

Patient descriptions consistent with *Musical Integration* outlined that music absorption can help to provide an escape from the reality of pain. Specifically, patients reported that music could be used to transport them out of their current experience and helped them to stop thinking about their or problems and to focus on something else. These descriptions from patients correspond with the idea that music helps to reduce pain because it is absorbing on a cognitive and emotional level (Bradshaw et al., 2012; Gold and Clare, 2013; Guetin et al., 2013; Finlay, 2014). Patients’ rich descriptions of musical integration suggest that full musical absorption as opposed to music listening is required to mediate the analgesic benefits of musical engagement. This highlights the importance of facilitating immersive music-listening experiences to support patient engagement with the music (Bradshaw et al., 2012). In order to maximize the likelihood that patients will become fully absorbed, it is important to consider the wider musical experience to reduce the presence of other major distractors (Brattico and Pearce, 2013; Lee, 2016). Also, additional strategies to enhance the music-listening experience, such as additional visual support (Chanda and Levitin, 2013) and an optimal listening environment should also be considered.

Patient descriptions related to the theme of *Cognitive Agency* encompassed active engagement and the importance of individuality which were related to a strengthened sense of self-identity and social connectedness. This is particularly important in chronic pain management contexts where people often experience low self-esteem and low self-efficacy due to diminished capacity as a result of having chronic pain (Jensen et al., 1991). This finding is in line with previous suggestions that personally significant music can be used to enhance an individual’s sense of cognitive agency, and that this in turn can assist with identity formation (Saarikallio et al., 2020). Many patients with chronic pain become disconnected from their social network and experience a reduction in their capacity to complete daily activities. At the same time people with chronic pain report that they sometimes feel trapped and as if their personal world is getting smaller. Music listening can be used to help patients maintain a sense of their personal identity (Saarikallio et al., 2020) and a sense of agency, when people are encouraged to choose their own music (Howlin and Rooney, 2021b; Howlin et al., 2022). Additionally, music listening is a relatively easy activity, and perceived as less effortful compared to other types of cognitive tasks, which could be beneficial when patients may have diminished cognitive resources available due to the experience of chronic pain.

### Patients’ music preferences

A secondary aim of this study was to assess patient preference for music based on the levels of energy in the music. The results demonstrate that patients rated unfamiliar low-energy music as significantly more enjoyable, with a significantly higher analgesic potential and significantly less irritating compared to unfamiliar high-energy music. Additionally, patients demonstrated different patterns of emotional responses to music with low values of energy compared to music with high values of energy. Low-energy music was rated as inducing significantly higher *Wonder*, *Transcendence*, *Tenderness*, *Peacefulness*, *Sadness*, and *Nostalgia* emotional responses compared to *High-Energy* music. These results contrast with the results found in some experimental settings, where participants did not demonstrate a clear preference for a particular music energy (Zhao and Chen, 2009). This may be due to the fact that patients with chronic pain are already in a state of relatively high arousal due to the presence of pain, which means they are more likely to become overloaded by unfamiliar high-energy music compared to healthy participants. In line with Berlyne’s (Berlyne, 1971) inverted-U theory in order to facilitate an enjoyable music-listening experience, music should not be too low in arousal or else it may be perceived as boring, and also should not be too high in arousal or it may be perceived as irritating. When we consider that patients have a tendency to choose music that is higher energy compared to experimenter music (Howlin and Rooney, 2021a), it is possible that the unfamiliar nature of this music may have made it particularly irritating due to a lack of context and meaning. We should consider the importance of optimizing arousal within the music-listening experience to ensure it is neither over stimulating or boring, based on each specific pain management context. It may be useful to introduce music that induces moderate levels of arousal to account for the possibility that chronic pain patients are already in an elevated state of arousal (Finlay and Rogers, 2015). In light of previous research and other findings from the present study, where possible participants will benefit from being given the option to choose their own music. This will allow them to select something that is optimal for their circumstances and serve to enhance their feelings of autonomy (Howlin and Rooney, 2021b).

### Strengths and limitations

An important aspect of this study is that it is the first study to investigate the cognitive mechanisms that mediate the analgesic benefits of music interventions with a specific clinical population, using a pre-defined theoretical model. This is important because as we can see from these results, patient responses to music can be quite different to responses to music from healthy controls. Further work with different patient groups is required to develop our understanding of the cognitive mechanisms underlying successful music interventions. Precise clarification of the boundaries between each factor, and the order in which they occur could be determined using a confirmatory factor analysis, with additional items included in more accessible language. Nonetheless, the high-level agreement from participants across the items suggests that these items and factors are a strong representation of the patients’ intention for analgesic music listening, which demonstrates that the CVM has reasonable external validity from chronic pain patients’ perspective. It is also important to note the benefits of using research methods that facilitate enhanced patient access to the study. In this study, we used online data collection methods to include patients from a range of geographical locations who may be unable to attend multiple hospital appointments and invited patients to complete the survey at a time and location that was convenient for them. A limitation of this study is that it does not evaluate the direct effects of music listening for chronic pain management. Instead, this study focuses on the factors that patients think are most important in mediating the analgesic effects of music listening. An additional consideration of this study is that it was completed in an individual context, whereas most pain management programs tend to be completed in a group setting. Given that the social context of music listening can influence music preferences (Hargreaves and North, 1999), future studies may wish to consider examining the influence of social context or group dynamics on music-listening choices, to identify if people with chronic pain would have different music preferences in a group setting.

### Implications

Until now the degree to which the CVM corresponds with chronic pain patients’ experience of music listening as a pain management strategy, was untested. Overall, this study suggests that chronic pain patients’ reasons for choosing music for pain management are broadly in line with the mechanisms outlined in the CVM (Automated Attention, Cognitive Agency, Meaning-Making, Musical Integration, and Cognitive Vitality). Chronic pain patients reported that the degree to which a song will elicit Musical Integration is the most important factor leading to subsequent analgesic benefits, but they also think that their specific music choices are an important component in achieving music analgesia. The role of motivation, and optimal arousal, was identified by participants as additional factors that need to be explored further. Qualitative responses from patients highlighted that Cognitive Agency was important because active engagement and individuality can help patients to strengthen their sense of self. This is particularly important for chronic pain management where people often experience low self-esteem and low self-efficacy due to diminished capacity, and pain management strategies that enhance the patient’s internal locus of control have been shown to be the most effective (Crisson and Keefe, 1988; Mitchell and MacDonald, 2006; Mitchell et al., 2007; Finlay, 2014). Additionally, patient descriptions highlighted that Musical Integration is important because a truly immersive music-listening experience can provide an escape from painful experiences. Pain management programs aim to support patients in developing self-management skills, which requires ongoing motivation on behalf of the patient. Music-listening interventions provide an opportunity to support patients on a daily basis, by encouraging them to engage in a personally meaningful and absorbing activity. Additional focus should also be placed on the best way to incorporate music interventions in a multi-disciplinary approach to psychology-based pain management programs. For example, the role of music interventions as a support to physiotherapy has yet to be explored. The introduction of music to pain management programs may facilitate ongoing motivation and participation by enhancing patient’s cognitive vitality. This may provide the basis for using music as a compliment to or therapeutic alternative to usual Cognitive Behavioral Therapy (CBT) based rehabilitation and maintenance for people with chronic pain.

## Data availability statement

The raw data supporting the conclusions of this article will be made available by the authors, without undue reservation.

## Ethics statement

The studies involving human participants were reviewed and approved by St. Vincent’s Hospital Research Ethics Board. Anonymous electronic informed consent for participation was provided by all of the participants who took part in the study in accordance with the national legislation and the institutional requirements.

## Author contributions

All authors CH, RW, PD, and BR contributed to the initial research design and write-up of the final manuscript. CH and RW were responsible for data collection. CH and BR were responsible for data analysis and interpretation. All authors contributed to the article and approved the submitted version.

## Funding

This project was supported by a research grant from the Irish Research Council (grant number GOIPG/2017/1006). The funding agency did not have any influence on the results or findings of the study.

## Conflict of interest

The authors declare that the research was conducted in the absence of any commercial or financial relationships that could be construed as a potential conflict of interest.

## Publisher’s note

All claims expressed in this article are solely those of the authors and do not necessarily represent those of their affiliated organizations, or those of the publisher, the editors and the reviewers. Any product that may be evaluated in this article, or claim that may be made by its manufacturer, is not guaranteed or endorsed by the publisher.
